# High and low dose of luzindole or 4-phenyl-2-propionamidotetralin (4-P-PDOT) reverse bovine granulosa cell response to melatonin

**DOI:** 10.7717/peerj.14612

**Published:** 2023-01-16

**Authors:** Wenju Liu, Zhihao Chen, Rui Li, Menghao Zheng, Xunsheng Pang, Aiyou Wen, Bing Yang, Shujuan Wang

**Affiliations:** 1 College of Life and Health Science, Anhui Science and Technology University, Fengyang, China; 2 Key Lab of Agricultural Animal Genetics, Breeding and Reproduction of Ministry of Education, Huazhong Agricultural University, Wuhan, China; 3 College of Animal Science, Anhui Science and Technology University, Fengyang, China; 4 Anhui Province Key Laboratory of Animal Nutritional Regulation and Health, Fengyang, China

**Keywords:** Melatonin, Luzindole, 4-P-PDOT, Hormonal synthesis, Gene regulation, Bovine, Granulosa cell

## Abstract

**Background:**

Communication between oocytes and granulosa cells ultimately dictate follicle development or atresia. Melatonin is also involved in follicle development. This study aimed to investigate the effects of melatonin and its receptor antagonists on hormone secretion, as well as gene expression related to hormone synthesis, TGF-*β* superfamily, and follicle development in bovine granulosa cells, and assess the effects of melatonin in the presence of 4-P-PDOT and luzindole.

**Methods:**

Bovine ovaries were collected from a local abattoir and follicular fluid (follicle diameter 5–8 mm) was collected for granulosa cell isolation and culture. Granulosa cells and culture medium were collected 48 h after treatment with melatonin at high dose concentrations (10^−5^ M) and low dose concentrations (10^−9^ M) in the absence/presence of 4-P-PDOT and luzindole (10^−5^ M or 10^−9^ M). Furthermore, the expression level of genes related to hormonal synthesis (*CYP11A1*, *CYP19A1*, *StAR,* and *RUNX2*), TGF-*β* superfamily (*BMP6*, *INHA*, *INHBA*, *INHBB,* and *TGFBR3*), and development (*EGFR*, *DNMT1A,* and *FSHR*) were detected in each experimental group by real-time quantitative PCR. In addition, the level of hormones in culture medium were detected using ELISA.

**Results:**

Both 10^−5^ M and 10^−9^ M melatonin doses promoted the secretion of inhibin A and progesterone without affecting the production of inhibin B and estradiol. In addition, both promoted the gene expression of *INHA*, *StAR*, *RUNX2*, *TGFBR3*, *EGFR,* and *DNMT1A*, and inhibited the expression of *BMP6*, *INHBB*, *CYP11A1*, *CYP19A1,* and *FSHR*. When combined with different doses of 4-P-PDOT and luzindole, they exhibited different effects on the secretion of inhibin B, estradiol, inhibin A, and progesterone, and the expression of *CYP19A1*, *RUNX2*, *BMP6*, *INHBB*, *EGFR,* and *DNMT1A* induced by melatonin.

**Conclusion:**

High and low dose melatonin receptor antagonists exhibited different effects in regulating hormone secretion and the expression of various genes in response to melatonin. Therefore, concentration effects must be considered when using luzindole or 4-P-PDOT.

## Introduction

A female animal’s follicle pool is established before and after birth (Chen et al., 2020). Subsequently, most of the follicles undergo atresia, and less than 1% of follicles eventually reach the preovulatory stage during the reproductive period (Kaipia & Hsueh, 1997). It is well known that cross talk with the hypothalamic–pituitary–gonadal (HPG) axis plays a crucial role in follicular development and folliculogenesis. FSH and LH play essential roles in folliculogenesis and determining ovulatory capacity (Craig et al., 2007; George, Dille & Heckert, 2011). Additionally, ovary granulosa cells are involved in modulating follicular development. Oocytes are surrounded by granulosa cells and their follicular growth and atresia are closely related to granulosa cell function (Jiang et al., 2003; Choi et al., 2011). Granulosa cells’ functional changes affect follicular growth and atresia (Matsuda et al., 2012; Li & Albertini, 2013; Wang et al., 2017a). The follicular development process is complex with many factors involved, and ultimately ends in the final stage of folliculogenesis. How to improve follicular development and the maturation of oocytes is still a problem that is worthy of attention. Previous studies have focused on granulosa cells’ role in follicular development.

Granulosa cells are the target of FSH and LH, which mainly regulate granulosa cell proliferation and functional differentiation and responds to stimulation via LH surge, thereby absorbing a large amount of fluid to expand the follicular antrum and acquire ovulatory capacity (Chen et al., 2020). On the other hand, ovary endocrine and local autocrine/paracrine systems also play an essential role in folliculogenesis. When accompanied by granulosa cells undergoing functional differentiation, the synthesis of estradiol, progesterone, inhibin, activin, and other hormones also change during follicular development. One of the major characteristics is that the estradiol level within the dominant follicles and potential ovulatory follicles is higher compared with that of subordinate follicles and atretic follicles (Ireland & Roche, 1982; Ireland & Roche, 1983). Estradiol synthesized by *CYP19A1* in granulosa cells is related to follicle maturation and is essential for follicle development, ovulation, and inhibiting granulosa cell apoptosis (Sahmi et al., 2019; Okamoto et al., 2016). In addition, inhibins, a negative feedback of FSH secretion, suppress the further development of follicles through the pituitary-gonadal system (Xu et al., 2020). In contrast, activin promotes the activity of FSH (Bilezikjian et al., 2006). Therefore, granulosa cell hormone secretion is also a key regulator that is essential in mediating ovarian follicle maturation and ovulation (Chowdhury et al., 2016). Communication between the granulosa cells and oocytes within follicles ultimately dictate follicle development or atresia, and the factors synthesized by granulosa cells are closely related to the follicle development process (Matsuda et al., 2012; Chang, Qiao & Leung, 2016; Kidder & Vanderhyden, 2010). BMP6, a member of the transforming growth factor beta (TGF-*β*) superfamily derived by granulosa cells, plays a critical role in follicular development and steroidogenesis (Lochab & Extavour, 2017; Otsuka, Moore & Shimasaki, 2001). Therefore, granulosa cells play important roles in modulating ovary physiological functions via local paracrine and autocrine mechanisms.

In addition to the above-mentioned factors, melatonin is another hormone produced by granulosa cells that has an important role in modulating follicular development. Melatonin is well known as a regulator of antioxidant activity, antiapoptosis, circadian rhythm, and other aspects of reproduction (Tamura et al., 2009; Rosen et al., 2012; Takada et al., 2012). Melatonin was initially considered to be derived from the pineal gland, but now research has shown that it can be widely produced by many tissues including the reproductive organs, granulosa cells, and oocytes (Tamura et al., 2009; Acuña Castroviejo et al., 2014). Melatonin as a hormone has a broad spectrum of sources synthesized through multiple cells. Interestingly, melatonin concentration in follicular fluid is higher than in serum and is positively correlated with increasing follicular diameter (Li et al., 2019; Tamura et al., 2013). These findings confirm the importance of melatonin in regulating reproductive functions, such as inducing oocyte maturation, protecting granulosa cells, stimulating the hormone secretion of granulosa cells, and promoting embryo development (Tamura et al., 2013; Tian et al., 2014; Wang et al., 2012; Wang et al., 2017b). Melatonin can also stimulate aromatase expression and estradiol production in human granulosa cells (Cheng et al., 2020). Moreover, melatonin induces progesterone production in human granulosa-lutein cells through the upregulation of *StAR* expression, and melatonin levels in human follicular fluid are positively correlated with progesterone levels in serum (Fang et al., 2019). Multiple enzymatic reactions are involved in the complex steroidogenesis process (Fang et al., 2019; Devoto et al., 1999), and melatonin plays a part in the process of producing steroid hormones, such as estradiol and progesterone (Cheng et al., 2020; Fang et al., 2019). However, the mechanism of melatonin’s mediation of steroid hormone secretion remains unclear, especially regarding different concentrations of melatonin within its receptor antagonists.

MT1 and MT2 are considered melatonin receptors and mediate melatonin function, and both MT1 and MT2 belong to G-protein-coupled receptors (Dubocovich & Markowska, 2005). Melatonin and MT1 signaling modulate hamster reproductive function (Prendergast, 2010), are involved in the downstream reaction of luteinizing hormone, take part in the luteinization of granulosa cells (He et al., 2016a), and further modulate bovine embryo development (Wang et al., 2014). In addition, melatonin and MT2 participate in porcine granulosa cell steroidogenesis (He et al., 2016b). Moreover, melatonin and MT2 can improve egg-laying rates by increasing hen’s serum estradiol and decreasing ovarian gonadotropin-inhibitory hormone receptor expression (Jia et al., 2016). Therefore, melatonin and its receptors, MT1 and MT2, are involved in regulating complex reproductive mechanisms. However, evidence has showed that MT1 and MT2 could also act as complements to mediate the function of melatonin. Melatonin modulates cell survival and apoptosis through interactions with MT1 and MT2 in spermatozoa (Espino et al., 2011), human leucocytes (Espino, Rodríguez & Pariente, 2013), and bovine granulosa cells (Wang et al., 2012). Further, melatonin targets MT1 and MT2 and is involved in regulating ovarian function and stimulating the progesterone production of granulosa cells (Dubocovich & Markowska, 2005; Wang et al., 2012).

Luzindole and 4P-PDOT are widely used to antagonize melatonin receptors, MT1 and MT2, and are considered the gold standards in the pharmacological research on melatonin receptors (Dubocovich et al., 1998; Liu et al., 2016; Browning et al., 2000). However, they exhibit different affinities for MT1 and MT2. The affinity of luzindole for MT2 is 25-fold higher than for MT1. Therefore, luzindole, a nonselective MT1 and MT2 antagonist, competitively blocks MT1 and MT2. 4P-PDOT has 1,300-fold higher affinity for MT2 than MT1, and 4P-PDOT is a selective MT2 antagonist that can competitively block MT2 (Dubocovich et al., 1997).

There have been numerous studies on melatonin regulating the functions of granulosa cells. However, the effects of different concentrations of melatonin and its receptor antagonists on mediating the hormone synthesis of bovine granulosa cells are still unclear. Therefore, this study explored whether granulosa cells exposed to different concentrations of melatonin and its receptor antagonists would experience alterations in hormone synthesis and the expression of related genes required for normal function. We aimed to further investigate the molecular adaptation of bovine granulosa cells under different concentrations of melatonin and its receptor antagonists by detecting gene expression related to hormonal synthesis (*CYP11A1*, *CYP19A1*, *StAR*, and *RUNX2*); TGF-*β* superfamily (*BMP6*, *INHA*, *INHBA*, *INHBB*, and *TGFBR3*); development (*EGFR*, *DNMT1A*, and *FSHR*); and the hormone section of progesterone, estradiol, inhibin A, inhibin B, and activin B.

## Material and Methods

### Bovine granulosa cell isolation and culture

Granulosa cell isolation and collection were carried out following our previously described protocol with minor revisions (Wang et al., 2012; Wang et al., 2017a; Wang et al., 2018; Wang et al., 2021). Bovine ovaries were collected from a local abattoir in Bengbu (Anhui, China) and sent back to the laboratory within three hours in a thermos cup. The obtained follicular fluid (follicle diameter 5–8 mm) was centrifugated at 1,500 rpm for 5 min and the cell pellets were collected and digested by 0.25% trypsin with 0.025% EDTA (Gibco, Grand Island, NY, USA) for 5 min. After being digested, the cell pellets were centrifugated again and dispersed in Dulbecco’s Modified Eagle Medium (DMEM) (Gibco, Grand Island, NY, USA) supplemented with 10% fetal bovine serum (FBS; Hyclone, Logan, UT, USA) and antibiotics including streptomycin (50 µg/ml), penicillin (50 IU/ml) (Pen-Strep, Invitrogen, Carlsbad, CA, USA), and plasmocin (25 µg/ml; Invitrogen, San Diego, USA). Finally, the separated cells were seeded into 60-mm cell culture dishes. The granulosa cells were finally cultured in an incubator at 37 °C and containing 5% CO_2_. The experimental protocols in this study were reviewed and approved by the Anhui Science and Technology University Institutional Committee on Animal Care and Use. Three independent repeats were performed for all experiments.

### Cell treatment

Melatonin with luzindole or 4-P-PDOT was diluted in DMSO to a concentration of 0.01 M and further diluted to 10^−5^ M and 10^−9^ M, respectively. The final concentration of DMSO was adjusted to 0.2% in all dilutions, and 0.2% DMSO was set as the control. Then, granulosa cells were distributed into four groups as follows: the control group (the untreated cells that were incubated with culture medium containing 0.2% DMSO), the melatonin group (the cells that were exposed to 10^−5^ M and 10^−9^ M melatonin treatment, respectively), the melatonin plus luzindole group (the cells that were pretreated with 10^−5^ M and 10^−9^ M luzindole for 30 min and then exposed to 10^−5^ M and 10^−9^ M melatonin, respectively), and the melatonin plus 4-P-PDOT group (the cells that were treated with 10^−5^ M and 10^−9^ M 4-P-PDOT for 30 min prior to 10^−5^ M and 10^−9^ M melatonin treatment, respectively). One day before treatment, 2 − 5 × 10^5^ cells were cultured in a 12-well plate to reach 70–80% confluence at the time of treatment, and the medium was replaced with fresh medium containing melatonin in the presence or absence of luzindole or 4-P-PDOT. The bovine granulosa cells were harvested 48 h after treatment. Three independent repeats were performed for all experiments.

### RNA extraction

Granulosa cells were cultured and treated with melatonin in the presence or absence of luzindole or 4-P-PDOT for 48 h, and then collected, respectively. The total RNA was extracted using RNAprep pure cell Kit (Tiangen, Beijing, China), quantified by using Nanodrop One (Thermo Fisher Scientific, Waltham, MA, USA) at 260 nm and stored at −80 °C until use. RNA was reverse-transcribed into first strand cDNA using a cDNA Synthesis Kit (Thermo Fisher Scientific, Waltham, MA, USA) with RNase-free DNaseI to remove the genome DNA.

### Real-time PCR

Granulosa cells collected 48 h after melatonin treatment with or without 4P-PDOT or luzindole were used to measure the gene expression related to hormonal synthesis (*CYP11A1*, *CYP19A1*, *StAR*, and *RUNX2*), TGF-*β* superfamily (*BMP6*, *INHA*, *INHBA*, *INHBB,* and *TGFBR3*), and development (*EGFR*, *DNMT1A,* and *FSHR*). Briefly, quantitative real-time PCR was run using LightCycler 480 SYBR Green I Master Mix on LightCycler 480 II Real-Time PCR System (Roche, Penzberg, Germany) according to our previously reported method (Wang et al., 2012; Wang et al., 2017a; Wang et al., 2018; Wang et al., 2021). The primer pairs designed for detection are listed in Table 1. The quantitative real-time PCR reactions included LightCycler 480 SYBR Green I Master Mix (5 µL), specific primer (0.5 µM for each primer), reverse transcribed cDNA (2 µL), and RNase and DNase-free water ddH2O (2 µL). Amplification obtained was as follows: 95 °C for 5 min, 40 cycles at 95 °C for 20 s, annealing at particular temperatures for 20 s, 72 °C for 20 s; and a melting curve analysis was performed from 65 °C to 95 °C to confirm specific PCR products. Normalization was performed using *β*-actin in each sample as a control. Finally, the expression levels of each target gene were analyzed using the 2^−ΔΔCT^ method (Livak & Schmittgen, 2001).

**Table 1:** Sequences of primer pairs for quantitative real-time PCR.

Gene	Forward Primer sequence (5′→3′)	Reverse Primer sequence (5′→3′)	Length
*CYP11A1*	ATGCTGGAGGAGACAGTGAACC	GCAGTAGAGGATGCCTGGGTAA	249
*CYP19A1*	CACCCATCTTTGCCAGGTAGTC	ACCCACAGGAGGTAAGCCTATAAA	78
*StAR*	GTG GAT TTT GCC AAT CAC CT	TTATTG AAA ACG TGC CAC CA	203
*RUNX2*	AAGGCAAGGCTAGGTGGAAT	AGAGGGGCACAGACTTTGAA	189
*DNMT1A*	ACGAATGGTGGATTGCTGGT	CACGTCTTCGTAGGTGGAGTC	197
*EGFR*	CACTCATGCTCTATGACCCTACC	CTCACCGATTCCTATTCCGTTAC	176
*BMP6*	TACGCTGCCAACTACTGTGAC	GATGGCGTTCAGTTTCGTG	153
*INHA*	GCACCCTCCCAGTTTCATCT	GGTTGGGCACCATCTCATACT	230
*INHBA*	GCAGTCGCACAGACCTTTCCT	CTCACAGTAGTTGGCGTGGTAGC	196
*INHBB*	CCTCATCGGCTGGAACGACTGG	TGGACATGGTGCTCAGCTTGGTG	114
*FSHR*	GAAGAAAGCAGGTGGATGGA	GGCAGAGGAAAACTCCGTTA	126
*TGFBR3*	ACTGTTGCCCCACCATAGAG	CCTGGAAATCTTAGCCCTCA	103
*β-actin*	CATCGGCAATGAGCGGTTCC	CCGTGTTGGCGTAGAGGTCC	145

### Western blot

Western blot was performed as previously described (Wang et al., 2017b; Wang et al., 2018; Wang et al., 2021). Granulosa cells were collected after 48 h of treatment, lysed in RIPA buffer (Thermo Fisher Scientific, Waltham, MA, USA), then denatured by boiling for 5 min with SDS-PAGE protein sample loading buffer, and frozen at −80 °C. The proteins were separated using 10% polyacrylamide gel electrophoresis and then transferred to polyvinylidene fluoride membrane (Millipore, Bedford, MA, USA). First, the membranes were incubated with mouse monoclonal antibody (Runx2 (1:300), StAR (1:300), Inhibin *β*-B (1:300), and *β*-actin (1:500) (Santa Cruz, Dallas, TX, USA)) and rabbit polyclonal antibody (CYP11A1 (1:500) and BMP6 (1:500) (Bioss, Woburn, MA, USA)). Later, the secondary antibody with HRP labeled (goat anti-rabbit or goat anti-mouse; 1:5000; Santa Cruz, Dallas, TX, USA) was used to incubate the membranes. Finally, the membranes were detected using an ECL detection kit (Bio-Rad Laboratories, Hercules, CA, USA).

### Endocrine secretion detection

To assess the hormone level in granulosa cells treated with melatonin combined with 4-P-PDOT or luzindole, the culture medium was collected 48 h after the granulosa cell treatment with melatonin, melatonin plus 4-P-PDOT, or melatonin plus luzindole at the concentrations of 10^−5^ M or 10^−9^ M. The cell culture supernatants were collected and centrifuged at 1000 × g for 15 min. Finally, the supernatants were frozen at −80 °C until use. The measurement of progesterone, estradiol, inhibin A, inhibin B, and activin B were carried out according to the manufacturer’s protocols of the bovine enzyme-linked immunosorbent assay (ELISA) kits (Shanghai Bogoo Biological Technology Co., Ltd, Shanghai, China). The sensitivity of estradiol, inhibin, and activin B kits was 1.0 pg/ml and progesterone was 0.1 ng/ml.

### Study design

Granulosa cells and culture medium were collected 48 h after granulosa cell treatment with melatonin at high dose concentrations (10^−5^ M) and low dose concentrations (10^−9^ M) in the absence/presence of 4-P-PDOT or luzindole. Melatonin was applied to the medium at 10^−5^ M and 10^−9^ M to further investigate the molecular adaptation of bovine granulosa cells under different concentrations of melatonin, as well as to confirm whether different concentrations of melatonin receptor antagonists modulated granulosa cell function induced by melatonin, and the diversity across different concentrations of melatonin receptor antagonists. Therefore, experimental groups were designed depending on the addition of 4-P-PDOT, luzindole, or melatonin: control (melatonin), melatonin plus 4-P-PDOT, or melatonin plus luzindole. Furthermore, the expression level of genes related to hormonal synthesis (*CYP11A1*, *CYP19A1*, *StAR,* and *RUNX2*), TGF-*β* superfamily (*BMP6*, *INHA*, *INHBA*, *INHBB,* and *TGFBR3*) and development (*EGFR*, *DNMT1A,* and *FSHR*) were detected in each experimental group by real-time quantitative PCR. In addition, we also detected the hormone level in granulosa cell culture medium treated with melatonin in the absence/presence of 4-P-PDOT or luzindole. Melatonin, 4-P-PDOT, and luzindole were first dissolved in DMSO, and then the final concentration of melatonin, 4-P-PDOT, and luzindole were diluted to 10^−5^ M or 10^−9^ M in the DMEM (Gibco, Grand Island, NY, USA). Moreover, the same concentration of DMSO acted as a control.

### Statistical analysis

All data were subjected to Statistical Analysis Systems (SAS Inc., Cary, NC, USA). Analysis of significant difference was performed and compared using one-way ANOVA with the General Linear Models Procedure, followed by Duncan’s multiple comparisons. Data were reported as mean ± SEM of triplicate experiments (*n* = 3). *P* < 0.05 was considered a significant difference.

## Results

### Effects of melatonin and its receptor antagonist supplementation on endocrine related gene expression

**Figure 1:**
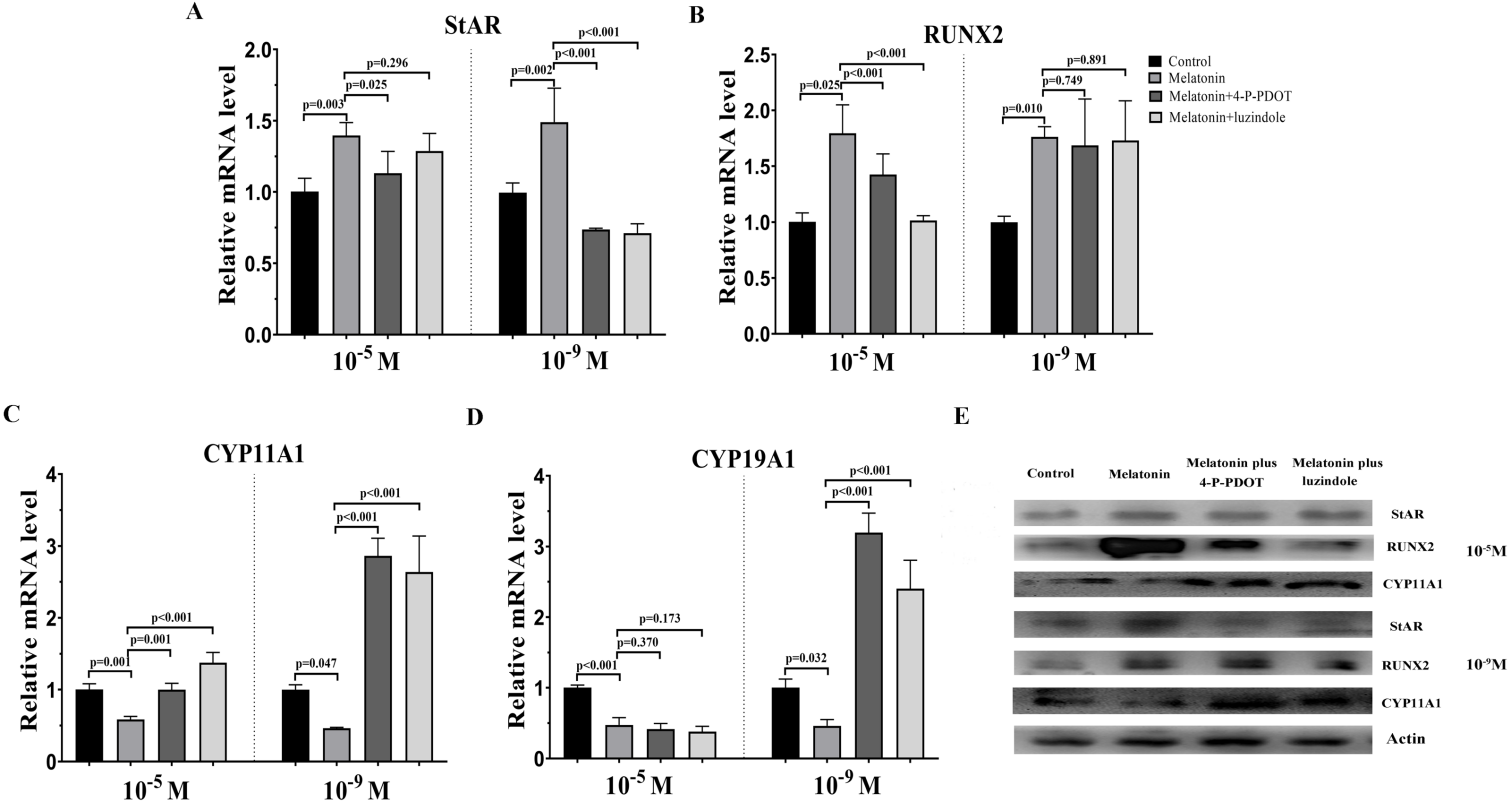
Effects of high dose (10^−5^ M) and low dose (10^−9^ M) melatonin and melatonin receptor antagonist supplementation on endocrine related gene expression (*StAR, RUNX2, CYP11A1,* and *CYP19A1*). The mRNA levels of *StAR* (A), *RUNX2* (B), *CYP11A1* (C), and *CYP19A1* (D) were examined by real-time PCR in granulosa cells 48 h after melatonin supplementation in the absence/presence of luzindole or 4-P-PDOT. (E) Protein abundance was detected by Western blot. The quantity of mRNA was normalized to that of *β*-actin. The statistical differences were performed using one-way ANOVA. *P* < 0.05 was considered significant difference. The experiment was repeated three times independently.

To assess the characteristics of bovine granulosa cells treated with high concentrations (10^−5^ M) and low concentrations (10^−9^ M) of melatonin in the absence/presence of its receptor antagonists, 4-P-PDOT or luzindole, hormone related gene (*StAR*, *CYP19A1*, *CYP11A,1* and *RUNX2*) expression was investigated by real-time PCR. Moreover, we also analyzed whether different concentrations of melatonin modulated granulosa cell function via its receptors. High concentrations (10^−5^ M) and low concentrations (10^−9^ M) of melatonin both significantly promoted the expression of *StAR* compared to control (Fig. 1A). However, high or low doses had different effects in the absence/presence of the receptor antagonists, 4-P-PDOT and luzindole. *StAR* expression was inhibited in the melatonin plus 4-P-PDOT group and melatonin plus luzindole group with low concentrations (10^−9^ M) compared to the melatonin group and control (Fig. 1A). Its expression was lower in the melatonin plus 4-P-PDOT group than the melatonin group (Fig. 1A), and there was no difference between the melatonin plus luzindole group and melatonin group at a high dose (10^−5^ M) (Fig. 1A, *p* > 0.05). The expression of *RUNX2* was significantly upregulated in the melatonin group, melatonin plus luzindole group, and melatonin plus 4-P-PDOT group compared to the control group (Fig. 1B), and no significant difference was observed after the melatonin treatment in the presence of 4-P-PDOT or luzindole at low concentrations (10^−9^ M) (Fig. 1B). Moreover, melatonin had a significantly higher expression of *RUNX2* compared to the control, and the *RUNX2* expression in melatonin plus 4-P-PDOT or luzindole groups was lower than that of the melatonin group at a high dose (10^−5^ M) (Fig. 1B). Melatonin significantly decreased the expression of *CYP11A1* while increasing its expression in melatonin treated with 4-P-PDOT or luzindole compared to the control at low concentrations (10^−9^ M) (Fig. 1C). Similarly, *CYP11A1* expression was significantly downregulated in the melatonin group compared to the control and upregulated in the melatonin plus 4-P-PDOT and melatonin plus luzindole groups compared to the melatonin group at high concentrations (10^−5^ M) (Fig. 1C). High concentrations (10^−5^ M) of melatonin in the absence/presence of 4-P-PDOT or luzindole significantly suppressed the expression of *CYP19A1* (Fig. 1D) and there was no difference across the melatonin group, melatonin plus 4-P-PDOT group, and melatonin plus luzindole group (Fig. 1D). In contrast, melatonin combined with 4-P-PDOT or luzindole significantly promoted the expression of *CYP19A1* and its expression was lower in the melatonin group at low concentrations (10^−9^ M) (Fig. 1D). Taken together, high concentrations (10^−5^ M) of melatonin receptor antagonists affected the melatonin’s ability to regulate the expression of *RUNX2* and *CYP11A1*, as well as its effect on modulating *StAR, CYP11A1,* and *CYP19A1* at low concentrations (10^−9^ M). Moreover, high concentrations (10^−5^ M) and low concentrations (10^−9^ M) of melatonin receptor antagonists did not affect the melatonin’s ability to regulate the expression of *CYP19A1* and *RUNX2,* respectively. Therefore, high and low concentrations of melatonin have the same effect on regulating the expression of *StAR*, *RUNX2*, *CYP11A1,* and *CYP19A1*. However, in the presence of 4-P-PDOT or luzindole, high and low concentrations of melatonin receptor antagonists exhibit different antagonistic effects against melatonin modulating endocrine related gene expression in granulosa cells.

### Effects of melatonin and its receptor antagonists’ supplementation on TGF-*β* superfamily related gene expression

**Figure 2:**
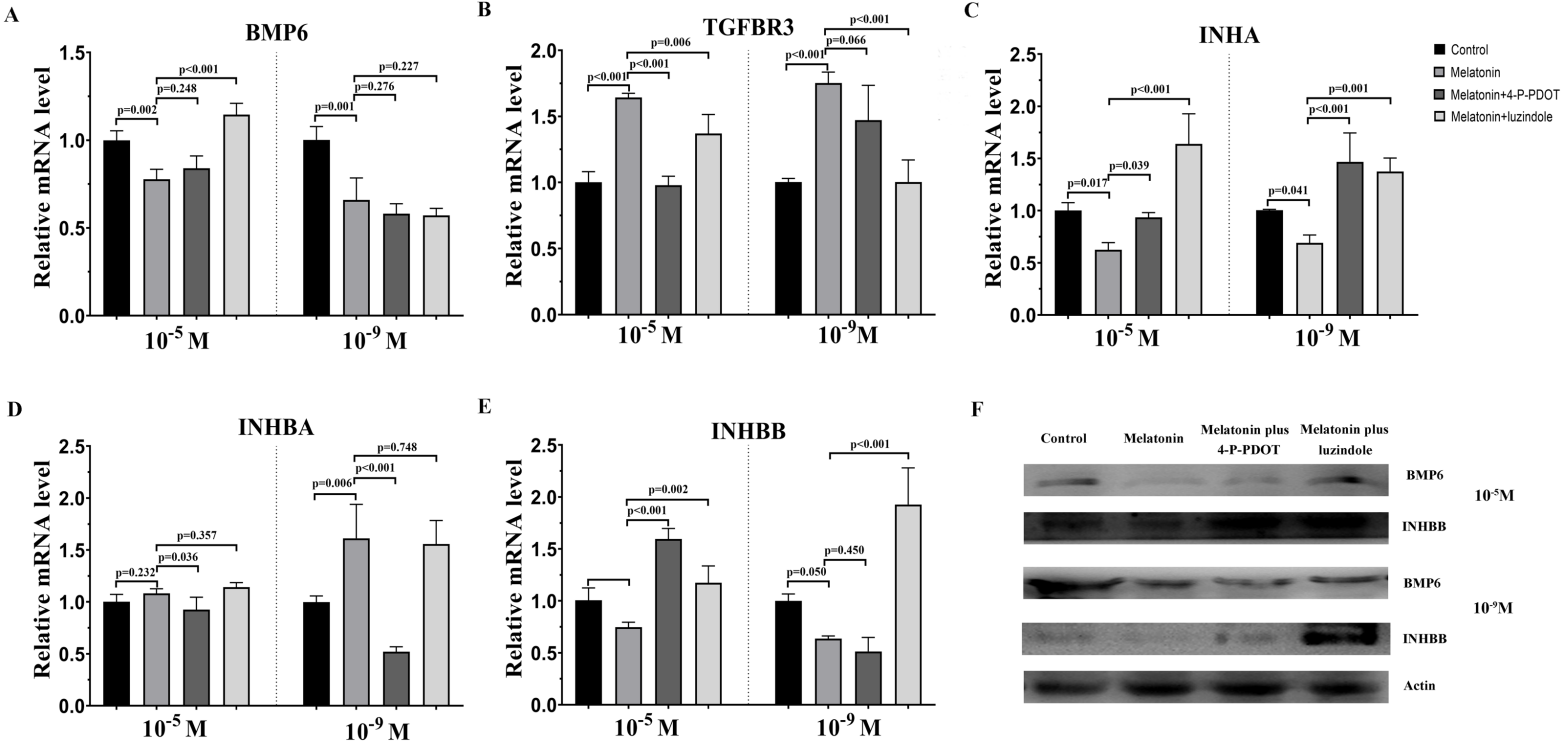
Effects of high dose (10^−5^ M) and low dose (10^−9^ M) melatonin and melatonin receptor antagonist supplementation on TGF-*β* superfamily related gene expression, including (*BMP6, TGFBR3, INHA, INHBA,* and *INHBB*). The mRNA abundance of *BMP6* (A), *TGFBR3* (B), *INHA* (C), *INHBA* (D), and INHBB (E) were examined by real-time PCR at 48 h after melatonin supplementation in the absence/presence of luzindole or 4-P-PDOT. (F) Protein abundance was detected by Western blot. mRNA abundance was normalized to that of *β*-actin. The statistical differences were performed using one-way ANOVA. *P* < 0.05 was considered significant difference. The experiment was repeated three times independently.

The TGF-*β* superfamily, including *BMP6*, *TGFBR3*, *INHA*, *INHBA,* and *INHBB,* related gene expression were assessed following melatonin treatment with or without 4-P-PDOT or luzindole. 10^−9^ M and 10^−5^ M melatonin both significantly inhibited the expression of *BMP6*, *INHA,* and *INHBB*, and induced the expression of *TGFBR3* (Fig. 2). However, the expression of *INHBA* was not changed in the 10^−5^ M melatonin group and improved in the 10^−9^ M group. The granulosa cells’ response to melatonin was also investigated when combined with 4-P-PDOT or luzindole. The expression of *BMP6* was not affected in the melatonin combined with 4-P-PDOT or luzindole compared to that melatonin in the 10^−9^ M dose (Fig. 2A). In the 10^−5^ M dose, there was no significant difference in the influence of 4-P-PDOT on the role of melatonin in the expression of *BMP6*, which was improved in the presence of luzindole (Fig. 2A). The changes in *TGFBR3* and *INHBB* were not significant in the granulosa cells treated with melatonin plus 4-P-PDOT when compared with melatonin in the 10^−9^ M dose (Fig. 2BE). In addition, 10^−9^ M of luzindole significantly reversed the effect of melatonin on increasing the expression of *TGFBR3* and inhibiting the expression of *INHBB* (Fig. 2BE). Conversely, *TGFBR3* and *INHBB* expression exhibited different characteristics in the 10^−5^ M dose, and they decreased and increased in the granulosa cells treated with melatonin when combined with 4-P-PDOT or luzindole, respectively, when compared to the melatonin group (Fig. 2BE). Moreover, both 10^−9^ M and 10^−5^ M of 4-P-PDOT and luzindole combined with melatonin promoted the expression of *INHA* compared to that in the melatonin group (Fig. 2C). This meant that 10^−9^ M and 10^−5^ M of 4-P-PDOT and luzindole had the same role in reversing the melatonin’s effect on *INHA* expression in granulosa cells. In regards to *INHBA*, only 10^−9^ M and 10^−5^ M of 4-P-PDOT could affect the melatonin’s role on *INHBA* expression (Fig. 2D), and luzindole did not mediate the expression of *INHBA* induced by melatonin (Fig. 2D). Therefore, both high doses (10^−5^ M) and low doses (10^−9^ M) of melatonin produced consistent effects on regulating TGF-*β* superfamily-related gene expression. High and low concentrations of melatonin receptor antagonists produced different effects.

### Effects of melatonin and its receptor antagonist supplementation on the development of related gene expression

**Figure 3:**
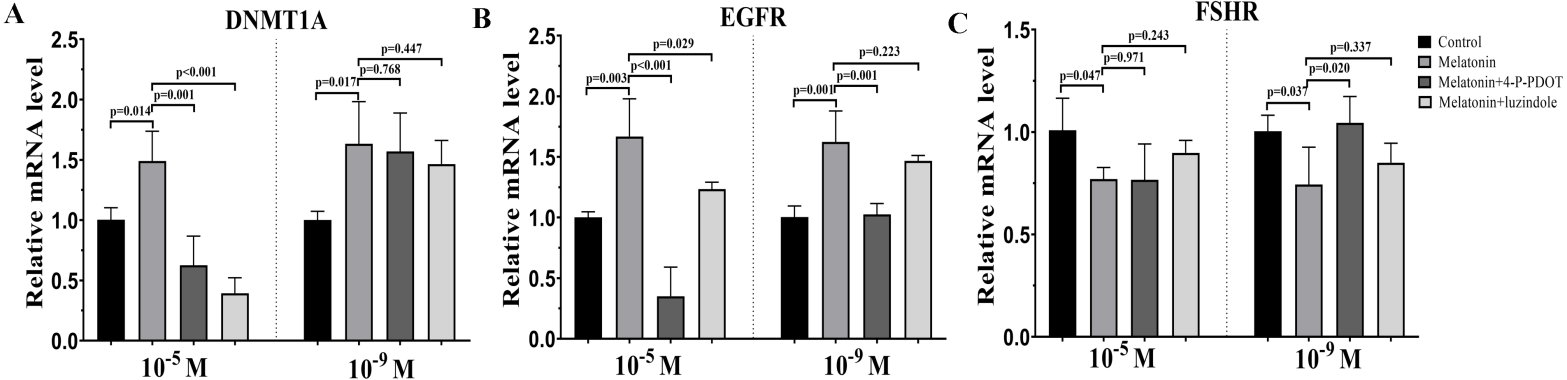
Effects of high dose (10^−5^ M) and low dose (10^−9^ M) melatonin and melatonin receptor antagonist supplementation on development related genes expression (*DNMT1A, EGFR,* and *FSHR*). The mRNA abundance of *DNMT1A* (A), *EGFR* (B), and *FSHR* were examined by real-time PCR 48 h after melatonin supplementation in the absence/presence of luzindole or 4-P-PDOT. mRNA abundance was normalized to that of *β*-actin. The statistical differences were performed using one-way ANOVA. *P* < 0.05 was considered significant difference. The experiment was repeated three times independently.

*DNMTA*, *EGFR,* and *FSHR* expression were also evaluated after melatonin treatment with or without 4-P-PDOT or luzindole. The expression of *DNMT1A* was significantly upregulated in the melatonin treatment group in the absence/presence of 4-P-PDOT or luzindole compared to the control (Fig. 3A), and there was no significant difference among the melatonin group, melatonin plus 4-P-PDOT group, and melatonin plus luzindole group in the low concentrations (10^−9^ M) (Fig. 3A). However, melatonin significantly increased the expression of *DNMT1A* when compared to the control group (Fig. 3A) and melatonin treatment in the presence of 4-P-PDOT or luzindole significantly decreased the expression of *DNMT1A* in the high concentrations (10^−5^ M) (Fig. 3A). Moreover, the expression of *EGFR* significantly increased in the melatonin group compared to the control while *EGFR* expression decreased in the melatonin plus 4-P-PDOT group compared to the melatonin group at concentrations of 10^−9^ M and 10^−5^ M (Fig. 3B). Melatonin plus luzindole did not affect the expression of *EGFR* at the low concentrations (10^−9^ M) (Fig. 3B) and downregulated the *EGFR* expression at the high concentrations (10^−5^ M) compared to the melatonin group (Fig. 3B). In regards to *FSHR*, melatonin significantly inhibited its expression (Fig. 3C), while there was no significant difference among the melatonin group, melatonin plus 4-P-PDOT group, and melatonin plus luzindole group (Fig. 3C) at high concentrations (10^−5^ M) (Fig. 3C). Only 4-P-PDOT could reverse the melatonin’s effect on inhibiting *FSHR* expression at the low concentrations (10^−9^ M) (Fig. 3C).

### Effects of melatonin and its receptor antagonist supplementation on t hormone secretions

**Figure 4:**
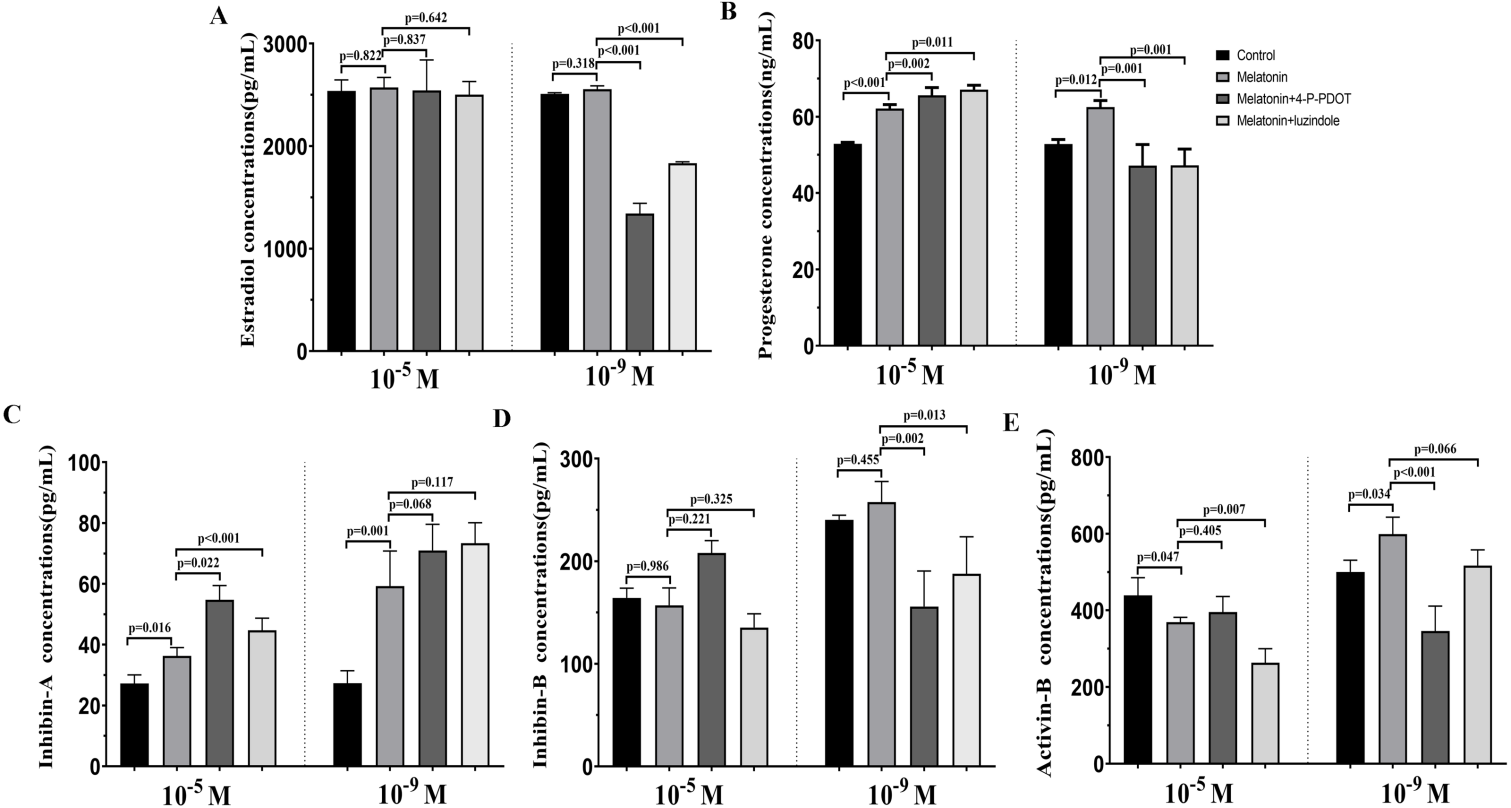
Effects of high dose (10^−5^ M) and low dose (10^−9^ M) melatonin and melatonin receptor antagonist supplementation on hormone secretions (estradiol, progesterone, Inhibin A, Inhibin B, and Activin B). Abundance of estradiol (A), progesterone (B), inhibin A (C), inhibin B (D) and activin B (E) were measured 48 h after melatonin supplementation in granulosa cell medium in the absence/presence of luzindole or 4-P-PDOT. The statistical differences were performed using one-way ANOVA. *P* < 0.05 was considered significant difference. The experiment was repeated three times independently.

The levels of estradiol, progesterone, Inhibin A, Inhibin B, and Activin B were measured 48 h after melatonin treatment in the absence/presence of 4-P-PDOT and luzindole. The concentration of estradiol was not altered after melatonin treatment compared to the control group (Fig. 4A) and significantly decreased in the melatonin plus 4-P-PDOT group and melatonin plus luzindole group compared to the melatonin group (Fig. 4A) at the low concentrations (10^−9^ M). Estradiol concentrations did not significantly differ after melatonin treatment in the absence/presence of 4-P-PDOT or luzindole (Fig. 4A) at the high concentrations (10^−5^ M). Progesterone levels were higher than that of the control group (Fig. 4B) but subsequently decreased following melatonin treatment in the absence/presence of 4-P-PDOT or luzindole compared to the melatonin group (Fig. 4B) at the low concentrations (10^−9^ M). In the high concentrations (10^−5^ M), progesterone levels were higher in the melatonin group than in the control and both melatonin treatments with 4-P-PDOT and luzindole significantly promoted progesterone levels compared with the melatonin group (Fig. 4B). Compared to the control group, Inhibin A abundance increased in the melatonin plus 4-P-PDOT group, melatonin plus luzindole group, and melatonin group compared to the control group in the low (10^−9^ M) and high concentrations (10^−5^ M) (Fig. 4C). Moreover, there was no significant difference across the melatonin plus 4-P-PDOT group, melatonin plus luzindole group, and melatonin group (Fig. 4C) in the low (10^−9^ M) concentrations, while Inhibin A abundance was higher in the melatonin plus 4-P-PDOT group and melatonin plus luzindole group than in the melatonin group at the low concentrations (10^−9^ M) (Fig. 4C). The concentrations of inhibin B showed no change in the melatonin group (Fig. 4D) at the low (10^−9^ M) and high concentrations (10^−5^ M). Both the melatonin plus 4-P-PDOT group and melatonin plus luzindole group significantly reduced the concentrations of inhibin B compared to the melatonin group (Fig. 4D) in the low concentrations (10^−9^ M). Inhibin B levels did not significantly differ after melatonin treatment in the absence/presence of luzindole compared to melatonin (Fig. 4D) in the high concentrations (10^−5^ M). Activin B was also tracked after melatonin supplementation in the absence/presence of 4-P-PDOT or luzindole (Fig. 4E). Activin B was indeed enhanced after melatonin supplementation and lower in the melatonin plus 4-P-PDOT group than in the melatonin group (Fig. 4E, *p* < 0.05) in the low concentrations (10^−9^ M). However, melatonin inhibited the activin B levels (Fig. 4E) in the high concentrations (10^−5^ M). Melatonin plus 4-P-PDOT did not affect the activin B level and melatonin plus luzindole significantly reduced the activin B level (Fig. 4E) compared to the melatonin group in the high concentrations (10^−5^ M).

## Discussion

**Figure 5:**
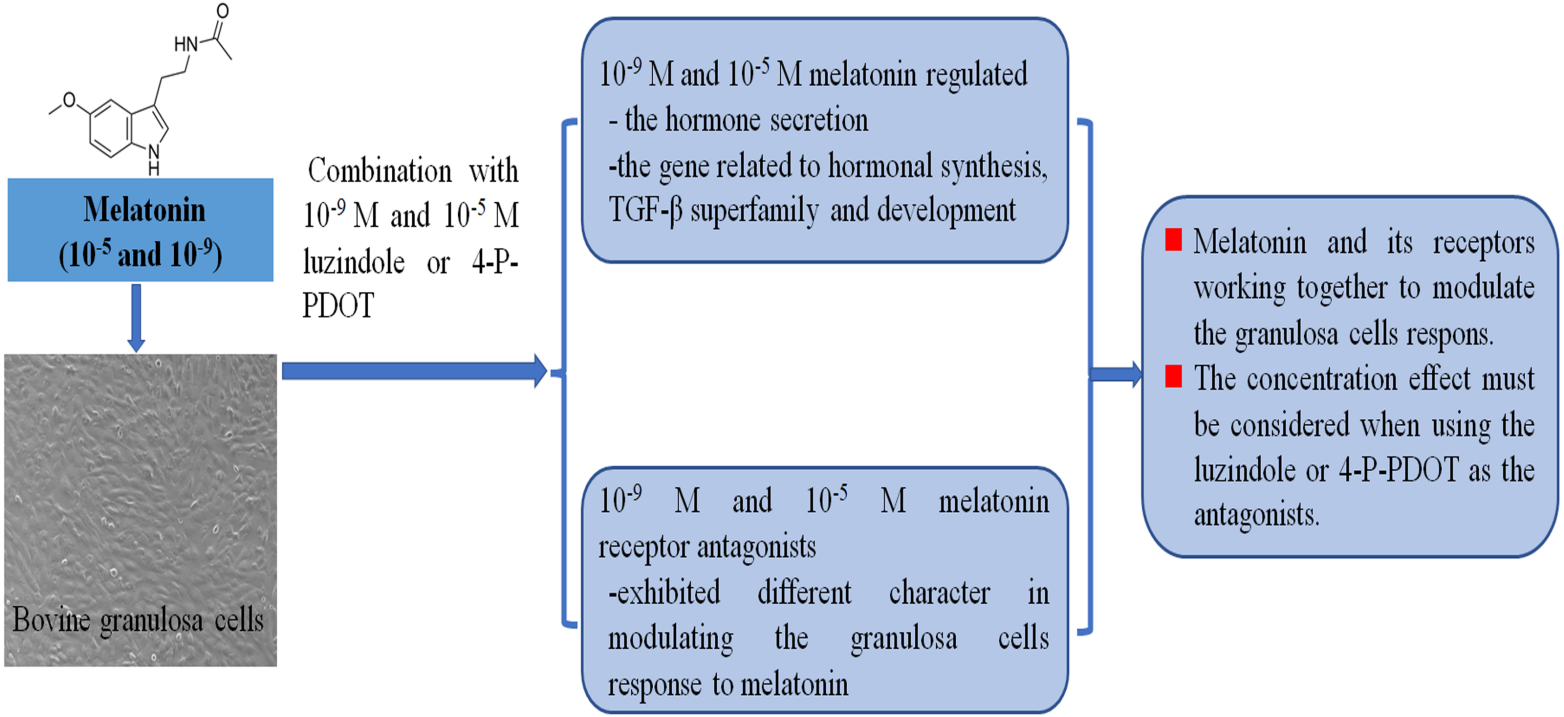
Melatonin and melatonin combined with its receptor antagonist mediated effects on bovine granulosa cells.

Follicular development, growth and maturation of oocytes, and ovulation are important events for reproduction. However, the follicular development process is complex, and many factors are involved in this process. Granulosa cells have exhibited important roles in follicular development and maturation of oocytes. One of the beneficial aspects is that many growth factors can be produced by granulosa cells and the hormones secreted by granulosa cells also mediate follicular development, such as estradiol, progesterone, inhibin, activin, and melatonin. The roles of melatonin in follicular development and maturation of oocytes have been well established in many animals. However, how melatonin mediates the hormone secretin remains unclear. In the present study, we investigated whether different concentrations of melatonin and its receptor antagonists alter both hormone synthesis and the related gene expression. The objective was to further explore the molecular adaptation of bovine granulosa cells on the hormone secretion of progesterone, estradiol, inhibin A, inhibin B, and activin B, and the gene expression related to hormonal synthesis (*CYP11A1*, *CYP19A1*, *StAR,* and *RUNX2*), TGF-*β* superfamily (*BMP6*, *INHA*, *INHBA*, *INHBB,* and *TGFBR3*), development (*EGFR*, *DNMT1A,* and *FSHR*) under different concentrations of melatonin and its receptor antagonists (Fig. 5).

Local ovary paracrine and autocrine mechanisms are involved in follicular development and many factors are secreted into the follicular fluid by granulosa cells in order to modulate the ovary physiological functions, especially follicular development and oocyte maturation (Chowdhury et al., 2016). The factors synthesized by granulosa cells provide communication between the granulosa cells and oocytes, which are closely related to oocyte maturation and also ultimately dictate follicle development (Matsuda et al., 2012; Chang, Qiao & Leung, 2016; Kidder & Vanderhyden, 2010). In the follicular fluid, the abundance of estradiol and progesterone are also crucial to promote follicular development and oocyte maturation. Estradiol and progesterone are synthesized in the granulosa cells by *CYP19A1* and CYP11A1, respectively (Pan et al., 2012). Additionally, StAR and RUNX2 are also involved in mediating the synthesis of steroidogenesis and progesterone (Fang et al., 2019; Park et al., 2010). However, it is poorly understood how melatonin affects the secretion of estradiol and progesterone by granulosa cells, as well as the effect of different dosages of melatonin with or without its antagonists. In this study, we found that both low doses (10^−9^ M) and high doses (10^−5^ M) of melatonin significantly promoted the secretion of progesterone and did not influence the synthesis of estradiol while stimulating the expression of StAR and RUNX2 and suppressing *CYP19A1* and *CYP11A1* expression. Melatonin induced progesterone production through the upregulation of StAR expression in human granulosa-lutein cells (Devoto et al., 1999), pregnant sow corpus luteum (Zhang et al., 2018), and bovine theca cells (Wang et al., 2019), which corresponded with the results of our study. Our previous study also indicated that 1,200 pg/ml of melatonin induced progesterone secretion and StAR and RUNX2 expression (Wang et al., 2018). Therefore, melatonin mediates the secretion of progesterone by stimulating StAR and RUNX2 expression. Moreover, *CYP19A1* and *CYP11A1* expression was not consistent with the production of estradiol and progesterone. In agreement with a previous study, *CYP11A1* had a downregulating expression pattern after melatonin treatment, in spite of a promotion in progesterone production, and StAR may be promoted in response to lower levels of *CYP11A1* (Zhen et al., 2014; Tanavde & Maitra, 2003). Therefore, the high concentration of progesterone secreted in granulosa cells by inducing melatonin acts as a negative feedback regulator of *CYP11A1* transcription (Wang et al., 2018; Pan et al., 2012; Zhen et al., 2014; Tanavde & Maitra, 2003). The estradiol level was not changed in granulosa cells in the presence of melatonin. However, a decrease in the expression of *CYP19A1* was observed after 48 h of melatonin treatment. A possible explanation might be that melatonin induced the secretion of estradiol in the first 24 h after melatonin treatment (unpublished data) and afterward acted as a negative feedback regulator of *CYP19A1* expression.

Both luzindole and 4-P-PDOT are melatonin receptor antagonists; however, they exhibit different role in inhibiting MT1 and MT2 receptors. Luzindole has antagonism to MT1 and MT2, but 4-P-PDOT is only a MT2 receptor antagonist (Wang et al., 2014; Emet et al., 2016). Curiously, there was a difference between the high and low concentrations of luzindole and 4-P-PDOT (10^−9^ M or 10^−5^ M) on influencing the effects of melatonin on hormone secretion and related gene expression. 10^−9^ M luzindole or 4-P-PDOT significantly inhibited the secretion of progesterone, decreased the expression of *StAR,* and increased the transcription of *CYP11A1* by blocking the effects of melatonin. This indicated that melatonin could mediate the progesterone secretion and related gene expression through MT1 and MT2. Previous studies indicated that melatonin stimulated *StAR* expression and progesterone production in bovine theca cells, human granulosa-lutein cells, and the corpus lutea of pregnant sows via MT1 and MT2 using the melatonin receptor antagonists, luzindole and 4-P-PDOT (Fang et al., 2019; Zhang et al., 2018; Wang et al., 2019). 10^−9^ M luzindole or 4-P-PDOT in combination with melatonin significantly decreased the level of estradiol and increased the expression of *CYP19A1*. This means that 10^−9^ M luzindole and 4-P-PDOT could change the effects of melatonin on estradiol secretion, but not the expression of *RUNX2* induced by melatonin. The probable reason for this is that the level of estradiol is negatively associated with *RUNX2* expression in human cumulus cells, and porcine and bovine granulosa cells (Wang et al., 2018; Zhen et al., 2014; Papamentzelopoulou et al., 2012). The 10^−5^ M luzindole and 4-P-PDOT exhibited different effects compared with the 10^−9^ M luzindole and 4-P-PDOT. 10^−5^ M luzindole and 4-P-PDOT altered the role of melatonin in the expression of *CYP11A1*, *StAR,* and *RUNX2*, and enhanced melatonin regulation of the secretion of progesterone without changing melatonin’s role in the secretion of estradiol. This suggests that luzindole or 4-P-PDOT could mediate the melatonin’s effect on steroid synthesis and related gene expression depending, in part, on MT1 and MT2. Meanwhile, the active effects of high doses of luzindole or 4-P-PDOT on MT1 and MT2 should be considered. A higher concentration of luzindole or 4-P-PDOT (100 nM and above) can activate the melatonin receptors by coupling to G protein with or without their ligand and exhibit a constitutively active form acting as an inverse agonist (Browning et al., 2000; Witt-Enderby & Dubocovich, 1996; Roka et al., 1999; Dubocovich & Markowska, 2005).

Communication between oocytes and granulosa cells can influence oocyte competence and quality, and the members of the TGF-*β* superfamily are responsible for this communication process and play an essential role in folliculogenesis (De Conto, Matte & Cunha-Filho, 2021). Among them, BMP-6 plays important roles in the growth of healthy follicles, dominant follicle selection, steroidogenesis, follicle atresia, and cell–cell communication between oocytes and granulosa cells (Lochab & Extavour, 2017; Otsuka, Moore & Shimasaki, 2001; Wu et al., 2019). Moreover, the abnormal expression of *BMP-6* alters granulosa cell function in peritoneal women (Wu et al., 2019). Furthermore, genetic deletion of the *Bmp6* gene in female mouse result in reduced litter size, lower ovulation rate, poor oocyte quality, and decreased fertilization rate (Sugiura, Su & Eppig, 2010). Inhibins and activins acting in an analogous manner regulate the production and secretion of follicle-stimulating hormone and control ovarian follicle development, inducing the proliferation, differentiation, apoptosis, and luteinization of granulosa cells in primary and secondary follicles (De Conto, Matte & Cunha-Filho, 2021; Da Broi et al., 2018; Xu et al., 2020). In this study, we found that both the 10^−9^ M and 10^−5^ M melatonin significantly promoted the level of inhibin A without affecting the abundance of inhibin B. However, 10^−9^ M melatonin increased the concentration of activin B while 10^−5^ M melatonin decreased the concentration of activin B. The expression of hormone related genes was also further elucidated after melatonin treatment. In accordance with the alternation of inhibin A and activin B, both the 10^−9^ M and 10^−5^ M melatonin significantly promoted the expression of *INHBA* and *TGFBR3*, except for *INHBA* expression in the 10^−5^ M melatonin. In addition, the expression of *INHA*, *INHBB,* and *BMP6* was significantly inhibited after 10^−9^ M and 10^−5^ M melatonin treatment. Previous research showed that the reduced expression of *INHA* accompanied by a down-regulated expression of *TGFBR3*, which may block inhibin-TGFBR3 signaling and therefore act as a negative feedback regulator of FSH abundance in the rat anterior pituitary cells (Han et al., 2013). Although *Inhbb* expression is up-regulated, the secretion of Inhibin B is still decreased, indicating the importance of their co-production (Wang et al., 2017b; Han et al., 2013). Interestingly, the fertility and ovulation rates are improved in sheep, cattle, and rats when the inhibin activity is attenuated using the inhibin vaccines (Takedomi et al., 2005; Han et al., 2008; Dan et al., 2016). In the local microenvironment of follicular fluid, the interaction of factors involved in mediating follicular development is complex, and the level of melatonin in follicular fluid is positively correlated with increasing follicular diameter (Li et al., 2019; Tamura et al., 2013). Therefore, melatonin regulating the expression of the *TGF*-*β* superfamily, especially the inhibin, maybe present a variety of patterns.

When combined with luzindole or 4-P-PDOT, the high (10^−5^ M) and low (10^−9^ M) doses played different roles in mediating the melatonin’s effect of regulating the secretion of inhibin A, inhibin B, and activin B, and the expression of *INHA*, *INHBA*, *INHBB*, *TGFBR3,* and *BMP6*. 10^−9^ M 4-P-PDOT mostly reversed these effects, significantly down-regulating *INHBA*, up-regulating *INHA* expression, not affecting the expression of *INHBB*, *BMP6,* and *TGFBR3*, and significantly decreasing inhibin B and activin B secretion. 10^−9^ M luzindole mostly influenced these effects, significantly increasing *INHBB* and *INHA*, decreasing *TGFBR3* expression, not affecting the expression of *INHBA* and *BMP6*, and significantly inhibiting inhibin B and activin B secretion. In addition, both 10^−9^ M 4-P-PDOT and luzindole significantly promoted the effects of melatonin on inducing the production of inhibin A. 10^−5^ M 4-P-PDOT mostly reversed the effects of melatonin on granulosa cells, significantly promoting *INHA* and *INHBB* and suppressing *INHBA* and *TGFBR3* expression, without affecting the expression of *BMP6*, and significantly increasing inhibin A secretion. Moreover, 10^−5^ M luzindole mostly showed antagonism to melatonin, significantly up-regulating *INHA*, *INHBB,* and *BMP6,* inhibiting *TGFBR3* expression, but not affecting the expression of *INHBA*, while significantly inhibiting activin B secretion, but not inhibin B secretion. Melatonin receptor antagonists exhibited different characteristics when blocking the melatonin role via luzindole and 4-P-PDOT. Consistent with the present results, our previous study found that MT1, MT2, or MT1 and MT2 working together could modulate the melatonin-dependent responses in bovine granulosa cells, particularly inhibin and activin levels, and therefore regulate follicular development using RNAi (Wang et al., 2017b; Wang et al., 2018). However, the concentration effects must be considered when using luzindole or 4-P-PDOT. Although these are the initial findings, the possible mechanism among inhibins, activins, melatonin, and melatonin receptor antagonists during follicular development needs to be further explored.

*DNMT1A*, *EGFR,* and *FSHR* play important roles in modulating granulosa cell function during follicular development. Both 10^−5^ M and 10^−9^ M melatonin significantly promoted the expression of *DNMT1A* and *EGFR* while decreasing the expression of *FSHR*. The level of 10^−9^ M 4-P-PDOT and luzindole did not affect the role of melatonin in promoting the expression of *DNMT1A,* and only 4-P-PDOT affected the melatonin’s effect on *EGFR* and *FSHR* expression. Moreover, 10^−5^ M 4-P-PDOT and luzindole altered the melatonin’s effect on regulating *DNMT1A* and *EGFR* without affecting *FSHR* expression. In agreement with the present study, melatonin significantly stimulated *DNMT1A* and *EGFR* expression involved in oocyte maturation, signal transduction, and epigenetic reprogram promoting bovine oocyte maturation (Tian et al., 2014). In addition, melatonin produced the same effects on *DNMT1A* expression in bovine embryonic development, sheep oocyte maturation, and *EGFR* in sheep cumulus cells (Tian et al., 2017; Wang et al., 2014). The presence of melatonin receptors and *FSHR* may be directly involved in melatonin and FSH promotion of the *in vitro* development of caprine follicles (Saraiva et al., 2011; Barros et al., 2013). The present study indicated that both the high and low concentrations of melatonin affected the expression of *DNMT1A*, *FSHR,* and EGFR. Moreover, the melatonin receptors, MT1 and MT2, were not the only signaling pathway mediating the melatonin effects on the regulation of granulosa cell signal transduction, epigenetic reprogram, and promotion of oocyte maturation.

## Conclusion

In this study, we demonstrated the effects of high and low levels of melatonin treatment on hormone secretion and gene expression related to hormonal synthesis, TGF-*β* superfamily, and development in bovine granulosa cells. Additionally, we further investigated the difference of high and low concentrations of melatonin receptor antagonists, 4-P-PDOT and luzindole, in affecting melatonin in granulosa cells. The results indicated that the high concentrations (10^−5^ M) and low concentrations (10^−9^ M) of melatonin could both modulate hormone synthesis of progesterone, inhibin A, inhibin B, and activin B in bovine granulosa cells. Furthermore, the specific responses were involved in modulating gene expression related to hormonal synthesis (*CYP11A1*, *CYP19A1*, *StAR*, and *RUNX2*), TGF-*β* superfamily (*BMP6*, *INHA*, *INHBA*, *INHBB,* and *TGFBR3*), and development (*EGFR*, *DNMT1A,* and *FSHR*) in granulosa cells. Our study also emphasized that high dose and low dose melatonin receptor antagonists exhibited different characteristics in regulating hormonal synthesis, the related genes of the TGF-*β* superfamily, and development when blocking the melatonin’s role via luzindole and 4-P-PDOT in granulosa cells. Therefore, the concentration effects must be considered when using luzindole or 4-P-PDOT. We aimed to understand the function of melatonin and its receptor antagonists on modulating granulosa cells physiological functions. Also, this study presents a potential mechanism of MT1, MT2, or MT1 and MT2 working together to modulate melatonin-dependent responses in bovine granulosa cells.

##  Supplemental Information

10.7717/peerj.14612/supp-1 Data S1 The expression level of genes and hormones levels at 48 h after melatonin supplementation in the absence/presence of luzindole or 4-P-PDOT

10.7717/peerj.14612/supp-2 Figure S1 Effects of high dose (10^−5^ M) and low dose (10^−9^ M) melatonin receptor antagonist supplementation on the endocrine related genes expression (*StAR*,* RUNX2*, *CYP11A1* and *CYP19A1*) The mRNA levels of *StAR* (A),* RUNX2* (B), *CYP11A1* (C) and *CYP19A1* (D) were examined by real-time PCR in granulosa cells at 48 h after luzindole or 4-P-PDOT supplementation. The quantity of mRNA was normalized to that of *β*-actin. The statistical differences were performed using one-way ANOVA. *P* < 0.05 was considered significant difference. The experiment was repeated three times independently.

10.7717/peerj.14612/supp-3 Figure S2 Effects of high dose (10^−5^ M) and low dose (10^−9^ M) melatonin receptor antagonist supplementation on TGF-*β* superfamily related genes expression, including (*BMP6*, *TGFBR3*, *INHA*, *INHBA* and *INHBB*) The mRNA abundance of *BMP6* (A),*TGFBR3* (B), *INHA* (C), *INHBA* (D) and *INHBB* (E) were examined by real-time PCR at 48 h after luzindole or 4-P-PDOT supplementation. mRNA abundance was normalized to that of *β*-actin. The statistical differences were performed using one-way ANOVA. *P* < 0.05 was considered significant difference. The experiment was repeated three times independently.

10.7717/peerj.14612/supp-4 Figure S3 Effects of high dose (10^−5^ M) and low dose (10^−9^ M) melatonin receptor antagonist supplementation on development related genes expression (*DNMT1A*, *EGFR* and *FSHR*) The mRNA abundance of *DNMT1A* (A), *EGFR* (B) and *FSHR* were examined by real-time PCR at 48 h after luzindole or 4-P-PDOT supplementation. mRNA abundance was normalized to that of *β*-actin. The statistical differences were performed using one-way ANOVA. *P* < 0.05 was considered significant difference. The experiment was repeated three times independently.

## Additional Information and Declarations

### Data Availability

The following information was supplied regarding data availability: The raw data are available in the Supplemental Files.

## References

[ref-1] Acuña Castroviejo D, Escames G, Venegas C, Díaz-Casado ME, Lima-Cabello E, López LC, Rosales-Corral S, Tan DX, Reiter RJ (2014). Extrapineal melatonin: sources, regulation, and potential functions. Cellular and Molecular Life Sciences.

[ref-2] Barros VR, Cavalcante AY, Macedo TJ, Barberino RS, Lins TL, Gouveia BB, Menezes VG, Queiroz MA, Araújo VR, Palheta RC, Leite Jr MC, Matos H (2013). Immunolocalization of melatonin and follicle-stimulating hormone receptors in caprine ovaries and their effects during in vitro development of isolated pre-antral follicles. Reproduction in Domestic Animals.

[ref-3] Bilezikjian LM, Blount AL, Donaldson CL, Vale WW (2006). Pituitary actions of ligands of the TGF-*β* family: activins and inhibins. Reproduction.

[ref-4] Browning C, Beresford I, Fraser N, Giles H (2000). Pharmacological characterization of human recombinant melatonin mt1and MT2 receptors. British Journal of Pharmacology.

[ref-5] Chang HM, Qiao J, Leung PC (2016). Oocyte-somatic cell interactions in the human ovary-novel role of bone morphogenetic proteins and growth differentiation factors. Human Reproduction Update.

[ref-6] Chen Y, Wang X, Yang C, Liu Q, Ran Z, Li X, He C (2020). A mouse model reveals the events and underlying regulatory signals during the gonadotrophin-dependent phase of follicle development. Molecular Human Reproduction.

[ref-7] Cheng J, Fang L, Li Y, Wang S, Li Y, Yan Y, Jia Q, Wu Z, Wang Z, Han X, Sun Y (2020). Melatonin stimulates aromatase expression and estradiol production in human granulosa-lutein cells: relevance for high serum estradiol levels in patients with ovarian hyperstimulation syndrome. Experimental & Molecular Medicine.

[ref-8] Choi J, Jo M, Lee E, Choi D (2011). Induction of apoptotic cell death via accumulation of autophagosomes in rat granulose cells. Fertility and Sterility.

[ref-9] Chowdhury I, Thomas K, Zeleznik A, Thompson WE (2016). Prohibitin regulates the FSH signaling pathway in rat granulosa cell differentiation. Journal of Molecular Endocrinology.

[ref-10] Craig J, Orisaka M, Wang H, Orisaka S, Thompson W, Zhu C, Kotsuji F, Tsang BK (2007). Gonadotropin and intra-ovarian signals regulating follicle development and atresia: the delicate balance between life and death. Frontiers in Bioscience.

[ref-11] Da Broi MG, Giorgi VSI, Wang F, Keefe DL, Albertini D, Navarro PA (2018). Influence of follicular fluid and cumulus cells on oocyte quality: clinical implications. The Journal of Assisted Reproduction and Genetics.

[ref-12] Dan X, Liu X, Han Y, Liu Q, Yang L (2016). Effect of the novel DNA vaccine fusing inhibin *α* (1-32) and the RF-amide related peptide-3 genes onimmune response, hormone levels and fertility in Tan sheep. Animal Reproduction Science.

[ref-13] De Conto E, Matte U, Cunha-Filho JS (2021). BMP-6 and SMAD4 gene expression is altered in cumulus cells from women with endometriosis-associated infertility. Acta Obstetricia et Gynecologica Scandinavica.

[ref-14] Devoto L, Christenson LK, McAllister JM, Makrigiannakis A, Strauss JF (1999). Insulin and insulin-like growth factor-I and -II modulate human granulosa-lutein cell steroidogenesis: enhancement of steroidogenic acute regulatory protein (StAR) expression. Molecular Human Reproduction.

[ref-15] Dubocovich ML, Markowska M (2005). Functional MT1 and MT2 melatonin receptors in mammals. Endocrine.

[ref-16] Dubocovich ML, Masana MI, Iacob S, Sauri DM (1997). Melatonin receptor antagonists that differentiate between the human Mel1a and Mel1b recombinant subtypes are used to assess the pharmacological profile of the rabbit retina ML1 presynaptic heteroreceptor. Naunyn-Schmiedeberg’s Archives of Pharmacology.

[ref-17] Dubocovich ML, Yun K, Al-Ghoul WM, Benloucif S, Masana MI (1998). Selective MT2 melatonin receptor antagonists block melatonin-mediated phase advances of circadian rhythms. The Federation of American Societies for Experimental Biology Journal.

[ref-18] Emet M, Ozcan H, Ozel L, Yayla M, Halici Z, Hacimuftuoglu A (2016). A review of melatonin, its receptors and drugs. The Eurasian Journal of Medicine.

[ref-19] Espino J, Ortiz, Bejarano I, Lozano GM, Monllor F, Garca JF, Rodrguez AB, Pariente JA (2011). Melatonin protects human spermatozoa from apoptosis via melatonin receptor- and extracellular signal-regulated kinase-mediated pathways. Fertility and Sterility.

[ref-20] Espino J, Rodríguez AB, Pariente JA (2013). The inhibition of TNF–induced leucocyte apoptosis by melatonin involves membrane receptor MT1/MT2 interaction. Journal of Pineal Research.

[ref-21] Fang L, Li Y, Wang S, Yu Y, Li Y, Guo Y, Yan Y, Sun Y (2019). Melatonin induces progesterone production in human granulosa-lutein cells through upregulation of StAR expression. Aging.

[ref-22] George JW, Dille E, Heckert LL (2011). Current concepts of follicle stimulating hormone receptor gene regulation. Biology of Reproduction.

[ref-23] Han L, Mao DG, Zhang DK, Liang AX, Fang M, Moaeen-ud Din M, Yang L (2008). Development and evaluation of a novel DNA vaccine expressing inhibin alpha (1-32) fragment for improving the fertility in rats and sheep. Animal Reproduction Science.

[ref-24] Han L, Wu C, Riaz H, Bai L, Chen J, Zhen Y, Guo A, Yang L (2013). Characterization of the mechanism of inhibin -subunit gene in mouse anterior pituitary cells by RNA interference. PLOS ONE.

[ref-25] He CJ, Ma T, Shi JM, Zhang ZZ, Wang J, Zhu K, Li Y, Yang M, Song Y, Liu G (2016a). Melatonin and its receptor MT1 are involved in the downstream reaction to luteinizing hormone and participate in the regulation of luteinization in different species. Journal of Pineal Research.

[ref-26] He Y, Deng H, Jiang Z, Li Q, Shi M, Chen H, Han Z (2016b). Effects of melatonin on follicular atresia and granulose cell apoptosis in the porcine. Molecular Reproduction and Development.

[ref-27] Ireland JJ, Roche JF (1982). Development of antral follicles in cattle after prostaglandin-induced luteolysis: changes in serum hormones, steroids in follicular fluid, and gonadotropin receptors. Endocrinology.

[ref-28] Ireland JJ, Roche JF (1983). Development of nonovulatory antral follicles inheifers: changes in steroids in follicular fluid and receptors for gonadotropins. Endocrinology.

[ref-29] Jia Y, Yang M, Zhu K, Wang L, Song Y, Wang J, Qin W, Xu Z, Chen Y, Liu G (2016). Melatonin implantation improved the egg-laying rate and quality in hens past their peak egg-laying age. Scientific Reports.

[ref-30] Jiang J, Cheung C, Wang Y, Tsang BK (2003). Regulation of cell death and cell survival gene expression during ovarian follicular development and atresia. Frontiers in Bioscience.

[ref-31] Kaipia A, Hsueh AJ (1997). Regulation of ovarian follicle atresia. Annual Review of Physiology.

[ref-32] Kidder GM, Vanderhyden BC (2010). Bidirectional communication between oocytes and follicle cells: ensuring oocyte developmental competence. Canadian Journal of Physiology and Pharmacology.

[ref-33] Li R, Albertini DF (2013). The road to maturation: somatic cell interaction and self-organization of the mammalian oocyte. Nature Reviews Molecular Cell Biology.

[ref-34] Li Y, Fang L, Yu Y, Shi H, Wang S, Guo Y, Suo Y (2019). Higher melatonin in the follicle fluid and MT2 expression in the granulosa cells contribute to the OHSS occurrence. Reproductive Biology and Endocrinology.

[ref-35] Liu J, Clough SJ, Hutchinson AJ, Adamah-Biassi EB, Popovska-Gorevski M, Dubocovich ML (2016). MT1 and MT2 melatonin receptors: a therapeutic perspective. The Annual Review of Pharmacology and Toxicology.

[ref-36] Livak KJ, Schmittgen TD (2001). Analysis of relative gene expression data using real-time quantitative PCR and the 2(-Delta Delta C(T)) method. Methods.

[ref-37] Lochab AK, Extavour CG (2017). Bone Morphogenetic Protein (BMP) signaling in animal reproductive system development and function. Developmental Biology.

[ref-38] Matsuda F, Inoue N, Manabe N, Ohkura S (2012). Follicular growth and atresia in mammalian ovaries: regulation by survival and death of granulosa cells. Journal of Reproduction and Development.

[ref-39] Okamoto A, Ikeda M, Kaneko A, Kishida C, Shimada M, Yamashita Y (2016). The novel pig *in vitro* maturation system to improve developmental competence of oocytes derived from atretic non-vascularized follicle. Biology of Reproduction.

[ref-40] Otsuka F, Moore RK, Shimasaki S (2001). Biological function and cellular mechanism of bone morphogenetic protein-6 in the ovary. The Journal of Biological Chemistry.

[ref-41] Pan Z, Zhang J, Lin F, Ma X, Wang X, Liu H (2012). Expression profiles of key candidate genes involved in steroidogenesis during follicular atresia in the pig ovary. Molecular Biology Reports.

[ref-42] Papamentzelopoulou M, Mavrogianni D, Dinopoulou V, Theofanakis H, Malamas F, Marinopoulos S, Bletsa R, Anagnostou E, Kallianidis K, Loutradis D (2012). Detection of RUNX2 gene expression in cumulus cells in women undergoing controlled ovarian stimulation. Reproductive Biology and Endocrinology.

[ref-43] Park ES, Lind AK, Dahm-Kahler P, Brannstrom M, Carletti MZ, Christenson LK, Curry TE, Jo M (2010). RUNX2 transcription factor regulates gene expression in luteinizing granulosa cells of rat ovaries. Journal of Molecular Endocrinology.

[ref-44] Prendergast BJ (2010). MT1 melatonin receptors mediate somatic, behavioral, and reproductive neuroendocrine responses to photoperiod and melatonin in Siberian hamsters (Phodopus sungorus). Frontiers in Endocrinology.

[ref-45] Roka F, Brydon L, Waldhoer M, Strosberg AD, Freissmuth M, Jockers R, Nanoff C (1999). Tight association of the human Mel(1a)-melatonin receptor and G(i): precoupling and constitutive activity. Molecular Pharmacology.

[ref-46] Rosen RB, Hu D, Chen M, McCormick SA, Walsh J, Roberts JE (2012). Effects of melatonin and its receptor antagonist on retinal pig-ment epithelial cells against hydrogen peroxide damage. Molecular Vision.

[ref-47] Sahmi F, Sahmi M, Gévry N, Sahadevan P, Allen BG, Price CA (2019). A putative protein-RNA complex regulates posttranscriptional processing of cytochrome P450 aromatase (CYP19A1) in bovine granulosa cells. Molecular Reproduction and Development.

[ref-48] Saraiva MVA, Celestino JJH, Araujo VR, Chaves RN, Almeida AP, Lima-Verde IB, Duarte AB, Silva GM, Martins FS, Bruno JB, Matos MH, Campello CC, Silva JR, Figueiredo JR (2011). Expression of follicle- stimulating hormone receptor (FSHR) in goat ovarian follicles and the impact of sequential culture medium on in vitro development of caprine preantral follicles. Zygote.

[ref-49] Sugiura K, Su Y, Eppig J (2010). Does bone morphogenetic protein 6 (BMP6) affect female fertility in the mouse?. Biology of Reproduction.

[ref-50] Takada L, Junior AM, Mingoti GZ, Balieiro JC, Cipolla-Neto J, Coelho LA (2012). Effect of melatonin on DNA damage of bovine cumulus cells during in vitro maturation (IVM) and on *in vitro* embryo development. Research in Veterinary Science.

[ref-51] Takedomi T, Kishi H, Medan MS, Aoyagi Y, Konishi M, Itoh T, Yazawa S, Watanabe G, Taya K (2005). Active immunization against inhibin improves superovulatory response to exogenous FSH in cattle. Journal of Reproduction and Development.

[ref-52] Tamura H, Nakamura Y, Korkmaz A, Manchester LC, Tan D, Sugino N, Reiter RJ (2009). Melatonin and the ovary: physiological and pathophysiological implications. Fertility and Sterility.

[ref-53] Tamura H, Takasaki A, Taketani T, Tanabe M, Kizuka F, Lee L, Tamura I, Maekawa R, Asada H, Yamagata Y, Sugino N (2013). Melatonin as a free radical scavenger in the ovarian follicle. Journal of Endocrinology.

[ref-54] Tanavde VS, Maitra A (2003). In vitro modulation of steroidogenesis and gene expression by melatonin: a study with porcineantral follicles. Endocrine Research.

[ref-55] Tian X, Wang F, He C, Zhang L, Tan D, Reiter RJ, Xu J, Ji P, Liu G (2014). Beneficial effects of melatonin on bovine oocytes maturation: a mechanistic approach. Journal of Pineal Research.

[ref-56] Tian X, Wang F, Zhang L, He C, Ji P, Wang J, Zhang Z, Lv D, Abulizi W, Wang X, Lian Z, Liu G (2017). Beneficial Effects of melatonin on the *in vitro* maturation of sheep oocytes and its relation to melatonin receptors. International Journal of Molecular.

[ref-57] Wang F, Tian X, Zhang L, Gao C, He C, Fu Y, Ji P, Li Y, Li N, Liu G (2014). Beneficial effects of melatonin on in vitro bovine embryonic development are mediated by melatonin receptor 1. Journal of Pineal Research.

[ref-58] Wang S, Liu B, Liu W, Xiao Y, Zhang H, Yang L (2017a). The effects of melatonin on bovine uniparental embryos development in vitro and the hormone secretion of COCs. PeerJ.

[ref-59] Wang S, Liu W, Pang X, Dai S, Liu G (2018). The mechanism of melatonin and its receptor MT2 involved in the development of bovine granulosa cells. International Journal of Molecular Sciences.

[ref-60] Wang S, Liu W, Wang L, Pang X, Yang L (2017b). The role of Melatonin receptor MTNR1A in the action of Melatonin on bovine granulosa cells. Molecular Reproduction and Development.

[ref-61] Wang S, Liu W, Wen A, Yang B, Pang X (2021). Luzindole and 4P-PDOT block the effect of melatonin on bovine granulosa cell apoptosis and cell cycle depending on its concentration. PeerJ.

[ref-62] Wang S, Liu W, Wu C, Ma F, Ahmad S, Liu B, Jiang X, Zhang S, Yang L (2012). Melatonin suppresses apoptosis and stimulates progesterone production by bovine granulosa cells via its receptors (MT1 and MT2). Theriogenology.

[ref-63] Wang X, Meng K, He Y, Wang H, Zhang Y, Quan F (2019). Melatonin stimulates STAR expression and progesterone production via activation of the PI3K/AKT pathway in bovine theca cells. International Journal of Biological Sciences.

[ref-64] Witt-Enderby PA, Dubocovich ML (1996). Characterization and regulation of the human ML1A melatonin receptor stably expressed in Chinese hamster ovary cells. Molecular Pharmacology.

[ref-65] Wu HC, Chang HM, Yi Y, Sun Z, Lin Y, Lian F, Leung P (2019). Bone morphogenetic protein 6 affects cell–cell communication by altering the expression of Connexin43 in human granulosa-lutein cells. Molecular and Cellular Endocrinology.

[ref-66] Xu H, Khan A, Zhao S, Wang H, Zou H, Pang Y, Zhu H (2020). Effects of inhibin A on apoptosis and proliferation of bovine granulosa cells. Animals.

[ref-67] Zhang W, Wang Z, Zhang L, Zhang Z, Chen J, Chen W, Tong D (2018). Melatonin stimulates the secretion of progesterone along with the expression of cholesterol side-chain cleavage enzyme (P450scc) and steroidogenic acute regulatory protein (StAR) in corpus luteum of pregnant sows. Theriogenology.

[ref-68] Zhen Y, Wang L, Riaz H, Wu J, Yuan Y, Han L, Wang Y, Zhao Y, Dan Y, Huo L (2014). Knockdown of CEBP*β* by RNAi in porcine granulosa cells resulted in S phase cell cycle arrest and decreased progesterone and estradiol synthesis. The Journal of Steroid Biochemistry and Molecular Biology.

