# Supplementary figures and images for: High and low dose of luzindole or 4-phenyl-2-propionamidotetralin (4-P-PDOT) reverse bovine granulosa cell response to melatonin
Source: PeerJ. 2023 Jan 16;11:e14612. doi: 10.7717/peerj.14612 (PMC9851050; doi:10.7717/peerj.14612)

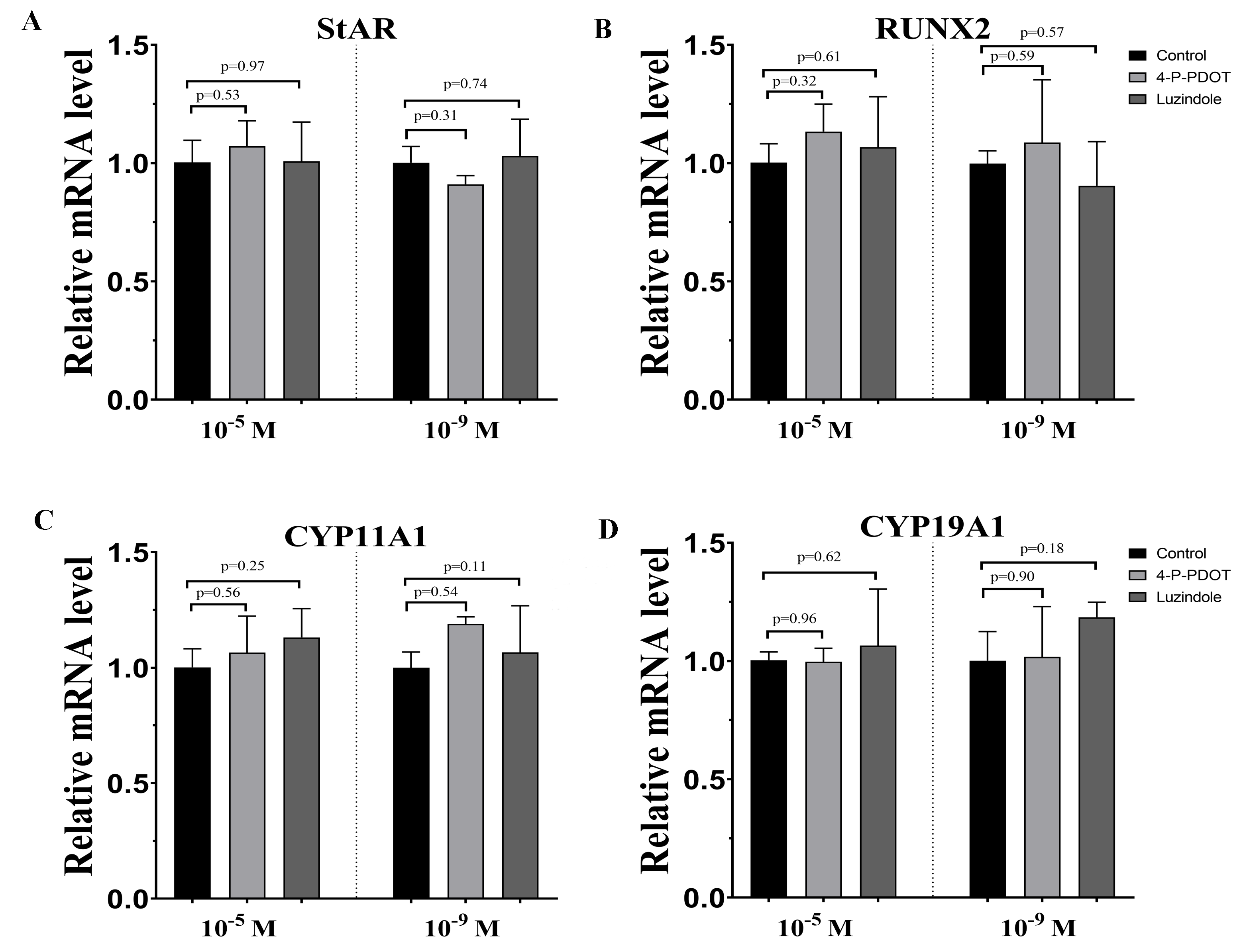

Supplement: Figure S1 — The mRNA levels of StAR (A), RUNX2 (B), CYP11A1 (C) and CYP19A1 (D) were examined by real-time PCR in granulosa cells at 48 h after luzindole or 4-P-PDOT supplementation. The quantity of mRNA was normalized to that of β-actin. The statistical differences were performed using one-way ANOVA. P < 0.05 was considered significant difference. The experiment was repeated three times independently. [file peerj-11-14612-s002.png]

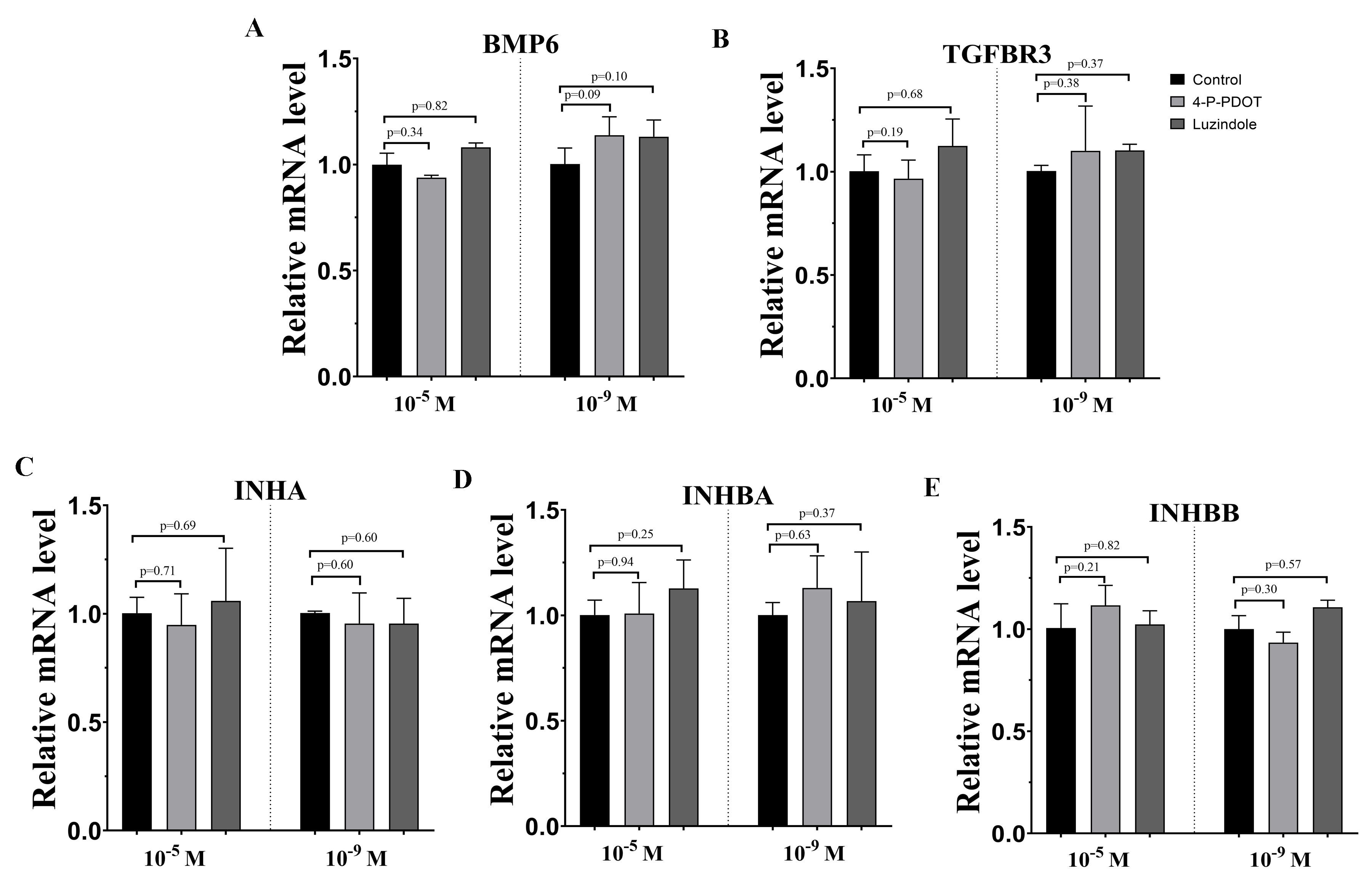

Supplement: Figure S2 — The mRNA abundance of BMP6 (A),TGFBR3 (B), INHA (C), INHBA (D) and INHBB (E) were examined by real-time PCR at 48 h after luzindole or 4-P-PDOT supplementation. mRNA abundance was normalized to that of β-actin. The statistical differences were performed using one-way ANOVA. P < 0.05 was considered significant difference. The experiment was repeated three times independently. [file peerj-11-14612-s003.png]

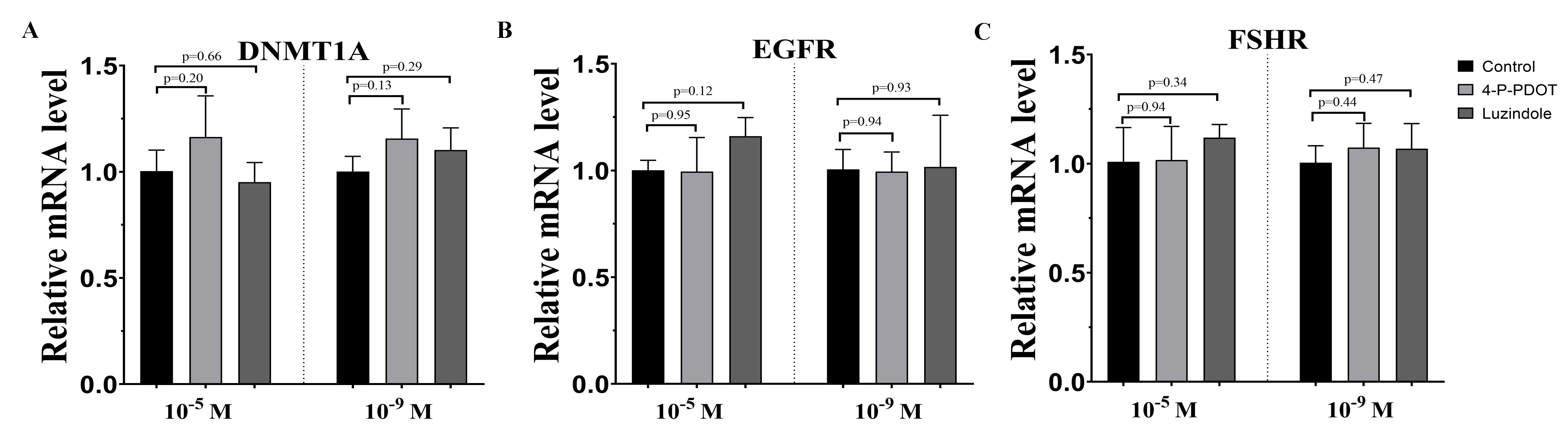

Supplement: Figure S3 — The mRNA abundance of DNMT1A (A), EGFR (B) and FSHR were examined by real-time PCR at 48 h after luzindole or 4-P-PDOT supplementation. mRNA abundance was normalized to that of β-actin. The statistical differences were performed using one-way ANOVA. P < 0.05 was considered significant difference. The experiment was repeated three times independently. [file peerj-11-14612-s004.png]
